# DDRGK1 in urine indicative of tubular cell injury in intensive care patients with serious infections

**DOI:** 10.15171/jnp.2016.13

**Published:** 2016-03-31

**Authors:** Dashurie Neziri, Sahra Pajenda, Rebecca Amuge, Aysegul Ilhan, Marlene Wewalka, Gregor Hörmann, Christian Zauner, Ludwig Wagner

**Affiliations:** ^1^Department of Internal Medicine III, Division of Nephrology and Dialysis, Medical University of Vienna, Vienna, Austria; ^2^Ugandan Christian University of Mbale, Mbale, Uganda; ^3^Division of Intensive Care 13H1, Medical University of Vienna, Vienna, Austria; ^4^Department of Laboratory Medicine, Medical University of Vienna, Vienna, Austria

**Keywords:** DDRGK1, C20orf116, Tubular cells, Acute kidney injury

## Abstract

*Background:* Acute kidney injury (AKI) is a life threatening condition. Despite intensive care treatment the occurrence cannot be predicted as very little indicators exist for direct measurement when tubular epithelial cell injury takes place. We therefore searched for novel peptide indicators expressed at intracellular level at the proximal kidney tubule for its appearance in urine samples.

*Objectives:* Establishing a test for urinary C20orf116 protein measurement.

*Patients and Methods:* Generation of immunoreagents against C20orf116 also named DDRGK1. These were used to measure its presence in urine collected at 8-24 hours interval in a prospective study from 99 ICU patients at 4-6 time points. These patients received therapy because of serious infection and were categorized into 4 groups.

*Results:* 1) Ten tested highly for C20orf116 undergoing AKI graded Failure or Loss (3210 ± 4268 ng/mL) according to RIFLE criteria, all requiring renal replacement therapy (RRT) out of them 9 died. 2) Six patients with pre-existing kidney disease developed AKI and required RRT but had much lower C20orf116 levels of (33 ± 19), two of them died. 3) In contrast, out of 11 patients undergoing AKI grade Risk or Injury, four tested positive for C20orf116 but to much lower extent (66 ± 43) who recovered fully. 4) Out of 72 patients 25 tested positive (18 ± 12 ng/mL) not fulfilling criteria of AKI but with serum creatinine (sCr) rises of 1.2-1.4 (n = 52). Healthy donors (n = 48) showed no detectable C20orf116 at any time point.

*Conclusions:* C20orf116 excretion was detectable more than 24 hours before sCr rise could be measured; high level seemed to indicate severity of organ failure.

**Implication for health policy/practice/research/medical education:**

Protein biomarker testing in urine for patients presenting with morbidities associated with high risk to undergo acute kidney injury (AKI) is a new option for early diagnosis of kidney stress und would therefore be helpful to mitigate injuries by early intervention strategies.

## 1. Background

Disease specific biomarker profiles have been established for various clinical conditions. This has enabled emergency departments and critical care units to rapidly respond by therapeutic strategies. Not only acute diagnosis of diseases but also monitoring of treatment response and disease progression has increasingly become a domain of biomarker follow up. Only few kidney specific biomarkers have been characterized up till now (1-7). Due to the fact that several proteins of unknown function had been structurally defined and its topology of expression has been elucidated through the human protein atlas (http://www.proteinatlas.org/) we have been motivated to look at kidney specific proteins which might appear in urine upon cellular disintegration in proximal kidney tubules the site of acute kidney injury (AKI). This represents an important topic as AKI accounts for a serious condition that affects many intensive care unit (ICU) patients with devastating consequences (8-11). Sepsis and septic shock remain the most common contributing factors in the development of AKI in ICU (8). Until recently, the development of AKI in sepsis patients was considered to be a condition of hemodynamic origin (12). However, recent data suggest that there is no impairment of the renal blood flow (RBF) in these patients. At the contrary, recent work has demonstrated that RBF remained normal or even increased during severe sepsis (13). Another mechanism that might be implicated in the development of septic AKI is apoptosis (14,15). Septic AKI accounts for close to 50% of cases of AKI in ICU (8). The mortality rate of AKI varies with the severity of AKI from 20.9% to 56.8%, compared with 8.4% among patients without AKI (16). Therefore, early prediction and detection of AKI is of vital importance to prevent its progression and thereby, possibly to improve its outcome (17). Consistent with this are the animal models which strongly suggest that early treatment is the key to prevent AKI (18,19).

The detection of AKI is based on serum creatinine (sCr) rise and reduction of urine output. However, this marker has limitations as a marker of glomerular filtration rate (GFR) because substantial kidney injury may have already occurred by the time sCr increases (20) and urine flow decreases. Therefore, reliable biomarkers that predict AKI before these two late indicators become evident have to be identified. In this respect, diverse urinary proteins have been evaluated as non-invasive indicators of AKI (1-3,5-7,21) and have already provided further insight.

Recently, we have cloned a novel protein, encoded by open reading frame 116 at chromosome 20 (known also as DDRGK1 and C20orf116), and demonstrated its abundant expression in the kidney and in the proximal tubular cells (22). In addition, it has been suggested that this protein is not a secreted moiety but rather resides at the endoplasmic reticulum (ER) due to its signal peptide (23). In our immunohistochemical analysis the protein was found in the lumen of some kidney tubules. It laid the foundation for the idea to test whether C20orf116 can be detected in human urine samples. Following initial experiments which showed C20orf116 in critically ill patient’s urine, we conducted a study comparing urine samples from healthy volunteers with critically ill patients. This was performed at our ICU in a prospective study and urinary C20orf116 was compared with routine kidney function tests and the clinical course of patients.

## 2. Objectives

Early diagnosis of kidney cell damage is of importance in medical care therefore we searched for novel urinary biomarkers indicative for tubular cell injury.

## 3. Patients and Methods

### 3.1. Urine collection

Catheter urine or clean catch urine samples were centrifuged (3000 rpm at 4°C for 10 minutes) to remove cell components and stored at -20°C for further use. The study design suggested urine sampling at admission/diagnosis and at 8-12, 24, 48, 72 and 96 hours following ICU treatment. All ICU patients were diagnosed with septicemia or severe infections (pneumonia). ICU patients were categorized according to RIFLE staging (R=risk, I=injury, F=failure, L=loss, E=end stage disease) into four groups 1) developing AKI grade F and L and requiring renal replacement therapy (RRT, n=10), 2) AKI grade I upon preexistent kidney disease with sCr >2.5 m/dl (n=6), 3) AKI grade R or I (n=11) and group 4) all remnants (n=72) not fulfilling AKI according to RIFLE criteria but with sCr rise 1.2-1.4 fold (n=52). Healthy individuals (n=48) collected 4 individual urine samples by clean catch over a period of at least 48 hours. The four time points of measurement were chosen in a way that from each urine donor a morning urine sample had been collected and the urine collection time interval was spread over a period of at least 48 hours.

### 3.2. Generation of C20orf116 overexpressing cell line

The full coding sequence of C20orf116 was cloned into the pMSCV plasmid and lentivirus particles were generated essentially as described earlier (24). The cell line C-643 was transduced and chemoselected using puromycine (4 ng/mL).

### 3.3. Direct ELISA

High binding E.I.A/R.I.A plates were coated over night at 4°C with 100 µl recombinant C20orf116 (12.5 ng/mL in PBS) generated as a His-tag fusion protein in Sf9 insect cells as described earlier (22). After washing the plate, 100 µl of antibody dilution series was added to each well and incubated for 1 hour at RT. Following a washing step with PBS containing 0.1% Tween 20, HRP-conjugated goat anti-mouse/rabbit Ab was added for Ab detection. Antibody binding was detected using the two component TMB substrate (KPL). This ELISA test has been used for generating antibodies against C20orf116.

### 3.4. Western blot analysis

Urine samples (30 µl) were loaded after denaturation in SDS sample buffer onto 10% SDS-PAGE gel. After electrophoresis, the proteins were electrotransferred onto a nitrocellulose using a semidry blotting device. Following the blocking step, the blotted membrane was consecutively exposed to the primary Ab (rabbit anti-C20orf116 Ab; diluted 1:2000, overnight at 4°C) and to the secondary Ab (HRP-conjugated goat anti-rabbit Ab; diluted 1:5000, 50 minutes at RT). Between each incubation step, the blotted membrane was washed in PBS containing 0.1% Tween 20. Antigen visualisation was performed by chemiluminescence using lumi-imager F1 (Roche).

### 3.5. Urine ELISA assay

High binding E.I.A/R.I.A plates (Costar, Cambridge, MA, USA) were coated with purified anti-C20orf116 IgG for 4 hours at RT or overnight at 4°C. After a brief wash, 50 µl urine was dispensed into the 50 µl of blocking solution (KPL) pre-applied onto the multi-well dish. The plate was then incubated at RT for 2 hours under constant shaking including a standard series at each plate. After a brief wash with PBS, biotinylated rabbit anti-C20orf116 IgG and biotinylated mAb A2 was applied (dilution 1:2000 in PBS/rabbit serum) and incubated for 1 hour at 37°C. Following a second washing step (4 washes with 300 µl PBS) NeutrAvidin (Stratagene^®^, La Jolla, CA, USA) (1/5000 in 0.5x blocking solution) was added and incubated at RT for 45 minutes. Wells were washed with PBS (4 times with 300 µl PBS) and developed with two component TMB substrate (KPL Inc. Gaithersburg, MD, USA) for 15 minutes at RT in the dark. The reaction was stopped by adding 100 µl 1M H_3_PO_4_ and read at 450 nm with the ELISA reader (Power wave_x_, Bio-tek instruments, Inc., USA) at 450 nm. The concentrations were then calculated according to the standard curve. All samples were analyzed in duplicate. In total 1280 samples had been available for testing.

### 3.6. Biotinylation of antibodies

Protein G-Sepharose eluted rabbit IgG or mAb A2 was dialyzed against PBS for 3 days at 4°C and at least three changes of dialysis fluid. D-Biotinoyl-ε-Aminocaproic Acid N-Hydroxysuccinimide Ester (Roche Diagnostics, Mannheim, Germany) was dissolved in DMSO (30 mg/ml) and biotin was added to the antibody solution under constant stirring in 15 fold molar excess. The biotinylation was carried out at RT over 2 hours and free biotin was removed from the antibody by gel filtration chromatography using Sephadex^®^G-25 (Boehringer Mannheim, Mannheim, Germany).

### 3.7. Ethical issues

The research followed the tenets of the Declaration of Helsinki. Informed consents were obtained. All patients took part in this study voluntary. Sample analysis was carried out by blinded investigators each in duplicate. The research was approved by the ethical committee of the Medical University of Vienna (ethic code: 721/2007).

### 3.8. Statistical analysis

The 5th to 95th percentile of C20orf116 values are demonstrated in Whiskers blots. Differences between multiple groups were calculated with SPSS using analysis of variance (ANOVA) with correction for post hoc analysis. Statistical significance was defined when *P*<0.05. The graphic representation was performed using GraphPad Prism (La Jolla, CA, USA).

## 4. Results

**Figure 1:**
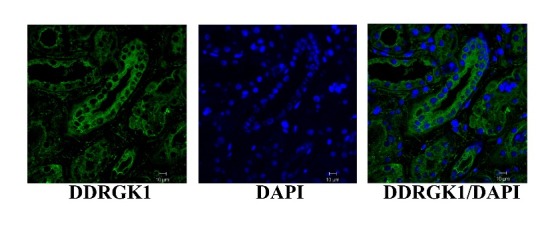
Immunostaining of kidney tubules: Specific proteinstaining mainly in the cytoplasmic compartment. The mAb A2 had been used for tissue staining and pictures were recorded using confocal microscopy.

High throughput gene expression analysis has generated extensive data sets on uncharacterized gene transcripts in various tissue types and disease processes. In order to obtain further information on till now undefined protein function we generated immunoreagents and established quantitative methods for mRNA and protein expression of C20orf116 also named Dashurin and DDRGK1. The presence of the protein was observed in kidney tubules in tissue sections. This has been established by immunofluorescence analysis of kidney cryosections (Figure 1). C20orf116 expression was also found in epithelia of urinary bladder and the urethra (data not shown). As already demonstrated in previous work the protein is not only expressed in kidney and urinary tract but is also found in the central nervous system, liver, placenta and heart (22,23,25).

### 4.1. ELISA quantification of urinary C20orf116

Motivated by a striking kidney tubule expression pattern as mentioned above we established a urinary ELISA method to measure C20orf116 in urine samples collected at 4-6 time points from intensive care patients (n=99) and we compared these with healthy donor urine which were collected at four different time points. Equivocally, all 48 healthy donors had no detectable level by our ELISA method. In the control group only healthy donors fully active in various professional backgrounds with sCr levels within the normal range were included.

**Figure 2:**
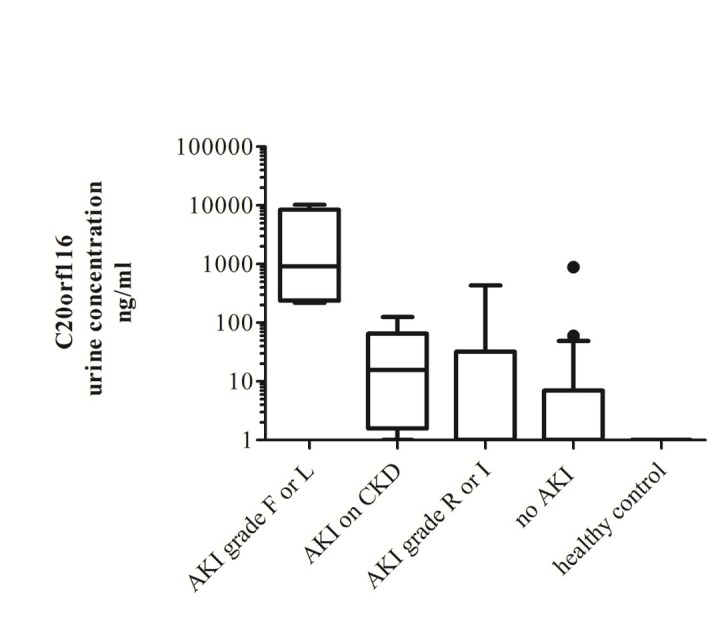
Urine C20orf116 measurement in ICU patients categorized into 4 groups: 1) RIFLE grade F-L requiring RRT without pre-existing kidney disease, 2) AKI on CKD requiring RRT, 3) RIFLE grade R and I 4) remnant 72 patients of whom 52 showed 1.2-1.4 fold sCr rise. The controls represent healthy donors.

**Figure 3:**
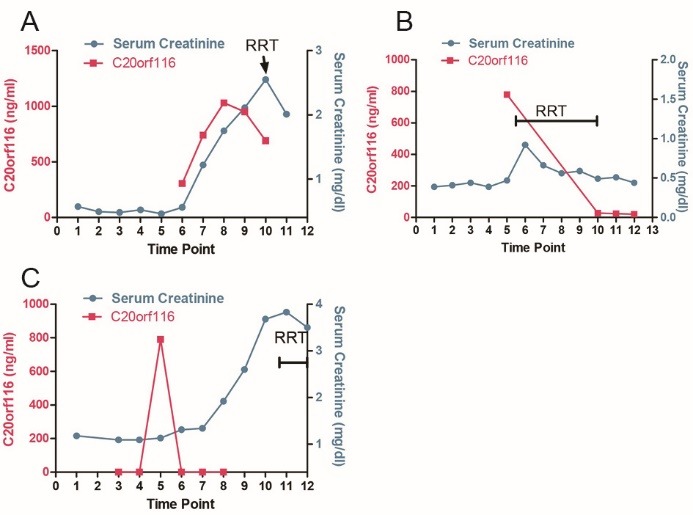
Urine C20orf116 comparing with sCr at the time point of study enrollment: A) patient with septic endocarditis receiving vancomycin in serum monitored therapeutic dosage undergoing AKI at unexpected conditions. B) Following septic shock high C20orf116 was followed by anuria and start of hemodialysis. This patient’s kidney function recovered entirely. C) A brisk episode of high C20orf116 in urine was followed by complete loss of kidney function. The patient died because of multiorgan failure.

In contrast to healthy controls 10 ICU patients tested high (3210±1350 ng/mL) for C20orf116 (Figure 2) at least 24 hours (Figure 3A) before sCr rise who had required RRT and were graded F or L according to RIFLE. Only one patient survived this disease episode. The patient who recovered is depicted at (Figure 3C). Six patients with pre-existing renal disease (sCr >2.5) developing AKI on chronic kidney disease (CKD) showed much lower level of C20orf116 (33±19 ng/mL) (Figure 2), these patients were included in the group AKI on CKD (Figure 2).

Eleven patients developed AKI grade R with C20orf116 (66±43 ng/ml) (Figure 2) and only 4 tested positive out of them. Finally out of 72 remaining patients who did not reach strict AKI criteria but developed 1.2-1.4 fold sCr rise 25 showed detectable C20orf116 (18±12 ng/ml) (Figure 2). All of them recovered. C20orf116 levels are statistically significantly different among the 5 groups (ANOVA, *P*<0.001). The post hoc test revealed again a statistically significant higher concentration of C20orf116 product in the group graded AKI, F or L when compared with other ICU groups and healthy controls (Tukey HSD, *P*<0.001).

**Figure 4:**
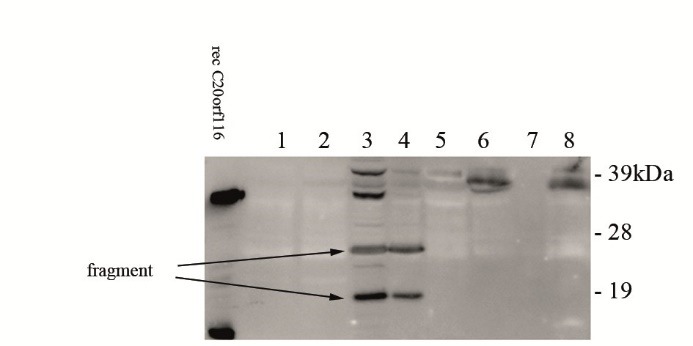
Immunoblotting urine of various ICU patients and control was loaded onto SDS-PAGE, transferred onto nitrocellulose and probed for variants of C20orf116. Various fragments as well as the entire size of the protein was detected depending on the patient. Included is in the first lane recombinant human C20orf116.

One of the patients in the group who did not reach AKI criteria showed high levels due to a non-explainable reason undergoing a sCr rise of 1.3 fold which was entirely reversible under intensive care treatment. She did not recover from infectious episodes for more than 5 months but was discharged from ICU after 1 month. Her kidney function tests stayed within the normal range.

Most importantly, in patients with previously intact kidney function high urinary levels of C20orf116 were detected 24-48 hours earlier before a rise in sCr could be observed (Figures 3A and B). Urine C20orf116 testing was carried out at 4-6 time points following diagnosis or admission to ICU as depicted in the methods section. As sample analysis was carried out in retrospect no further samples were available for testing at the time-point of C20orf116 measurement. SCr values were taken from the hospital routine data and used together with fluid output and disease records to evaluate grading and course of AKI.

Levels of C20orf116 in urine seemed to predict severity of kidney injury (Figure 2) and preceded sCr rises by 12-48 hours (Figure 3A-C) but high levels in some of the patients were still found when sCr returned to normal range and urine output increased.

### 4.2. Immunoblot characterization of urinary C20orf116

Earlier work has demonstrated that the DDRGK1 protein migrates at the SDS-PAGE at about 40 kDa (22,23) which is higher than the predicted molecular size would be expected. This was not only our observation but has also been described by Wu et al (23). Assuming that the urinary C20orf116 would not represent a physiological release product but rather a fragment which is liberated upon cell injury we performed multiple immunoblots of lyophilized urine samples and urine with high C20orf116 level according to ELISA tests derived from various ICU patients. A representative picture is demonstrated at Figure 4, various fragments of C20orf116 have been found in urine. The fragments seen at the blot were dependent on the antibody used for immunodetection of the urinary protein. In each experiment recombinant C20orf116 was included. The antibody used for immunoblotting was also essential for our ELISA testing and was included in each of the tests.

### 4.3. Tissue culture experiments

Although all data pointed towards detectability of the protein upon cellular breakdown we intended to assure that C20orf116 would not be a secretion product. Therefore we generated an over-expressing cell line as indicated in the methods section and tested whether the protein was detectable in tissue culture supernatant or not. Secretion of detectable amount could be excluded whereas intracellular protein was abundant which confirms data obtained by another group (23).

## 5. Discussion

In this study we present data from appearance of C20orf116 in body fluids such as urine in episodes of critical conditions in intensive care patients. This has been made possible after generating specific immunoreagents (polyclonal and monoclonal antibodies) against this protein. Using the newly established ELISA technique with these antibodies, we demonstrate that patients develop C20orf116-uria days or hours before sCr starts rising and oliguria develops. The idea to test C20orf116 in ICU patients was created following the observation that C20orf116 can be visualized in the lumen of kidney tubules by immunolocalization in tissue sections. Furthermore, it had been striking that especially the proximal kidney tubular cells (http://www.proteinatlas.org), the predilection site of tubular injury in AKI, has highest C20orf116 expression levels within this organ. It has been demonstrated recently that this protein forms complexes with the cell cycle check point kinase Cdk5rap3 and resides at the ER with its N-terminal hydrophobic signal peptide like structure (23). This described intracellular topology suggested that this protein does not represent a secreted molecule under physiological conditions at least in kidney epithelial cells as demonstrated in our work or in Hela cells as described previously (23). In consequence, our data point towards cellular disintegration which is then followed by release of this protein into the urine. To confirm these results we performed overexpression experiments and measurement in the tissue culture supernatant which revealed negative results confirming earlier data (23). In addition our observation of not detectable levels of C20orf116 in urine with the ELISA test of healthy individuals is further confirmatory that the protein fulfils its physiological function as a genuine intracellular protein. It therefore has to be assumed that appearance of C20orf116 in urine at high quantities is indicative for disintegration of cells at the tubular epithelium.

As all ICU patients were tested from urine obtained from a catheter placed through the urethra into the urinary bladder it has to be assumed that minor lesions of the urinary bladder do not contribute much to the level of C20orf116 in urine.

Taking into consideration that various other parameters such as IL-18, NGAL cystatin C (1,2,4,5,21) have been associated with AKI we have continuously attempted to search for other kidney born parameters which can be easily detected in urine before irreversible damages occur. We have now found one which seems to provide promising results especially as an early marker. One serious obstacle of this test represented the precipitation of recombinant protein upon freezing which had to be used for the standard curve. This problem could be resolved by dissolving the protein in 7M urea and then diluting it in urine. The urinary matrix seems to keep the protein better accessible to immunoreagents than any buffer used for recombinant protein solubilization.

Having demonstrated that fragments of C20orf116 might not be detected in all of the various samples we have been motivated to generate monoclonal antibodies against C20orf116 (aa 28-314). However, these antibodies did not prove usable in sandwich capture ELISA but together with the polyclonal antibody these might provide greater sensitivity in further experiments. Therefore polyclonal rabbit antibodies had to be produced for clinical specimen testing. These detected various fragments which originate from degradation during cell fragmentation and urine flow. Contamination with cells can be excluded due to the fact that each urine sample had been centrifuged as indicated in the methods section prior to freezing.

The observation that a subgroup of ICU patients with minor changes in sCr not fulfilling criteria of overt AKI show C20orf116 secretion in urine might indicate that tubular cell injury might occur silently not detectable by routine clinical parameters. In this direction observations by others showed that even minor changes in sCr while under ICU treatment will be of relevance for the further life span and patient outcome (26).

## 6. Conclusions

In conclusion we demonstrate that this novel protein expressed highly at the proximal kidney tubule might be promising to detect kidney damage which might be induced by potential nephrotoxic agents such as antibiotics and contrast agents and endogenous conditions at serious infections. However, most likely it will have to be combined with other novel markers such as Timp 2 and IGFBP 7 (5), KIM-1, neprilysin (27) or MIOX and others already or not yet characterized.

## Limitations of the study

Antibodies which are necessary for such measurement are not commercially available, furthermore the protein might be a too sensitive marker as a certain patient group presented with detectable levels without manifestation of any AKI stage**.** Comparison with other available biomarkers was beyond the borders of this study.

## Acknowledgments

Heinz Regele provided normal kidney tissue from tumor nephrectomy. D. Neziri received a scholarship from Astute Medical. We thank Panagiota Patsiou who helped to revise the manuscript.

## Authors’ contribution

DN was responsible for antibody generation and purification as well as sample analysis, SP helped in sample analysis and manuscript preparation and data recording, AR helped in laboratory work, AI performed statistical analysis, GH generated virus particles, MW was responsible for ICU patient care and sample collection, CZ supported the work as chief of the ICU and helped to enroll patient in the study, LW guided the study helped in ELISA development sample analysis and manuscript writing.

## Conflicts of interest

The authors declared no competing interests.

## Funding/Support

This study was in part the doctoral thesis of Dashurie Neziri. She was supported by a scholarship from Astute Medical. The department’s research budget paid for all chemicals and plastic ware used in the study.

## References

[1] Aregger F, Uehlinger DE, Witowski J, Brunisholz RA, Hunziker P, Frey FJ (2014). Identification of IGFBP-7 by urinary proteomics as a novel prognostic marker in early acute kidney injury. Kidney Int.

[2] Gaut JP, Crimmins DL, Ohlendorf MF, Lockwood CM, Griest TA, Brada NA (2014). Development of an immunoassay for the kidney-specific protein myo-inositol oxygenase, a potential biomarker of acute kidney injury. Clin Chem.

[3] Han WK, Bailly V, Abichandani R, Thadhani R, Bonventre JV (2002). Kidney injury molecule-1 (KIM-1): a novel biomarker for human renal proximal tubule injury. Kidney Int.

[4] Herget-Rosenthal S, Marggraf G, Husing J, Goring F, Pietruck F, Janssen O (2004). Early detection of acute renal failure by serum cystatin C. Kidney Int.

[5] Kashani K, Al-Khafaji A, Ardiles T, Artigas A, Bagshaw SM, Bell M (2013). Discovery and validation of cell cycle arrest biomarkers in human acute kidney injury. Crit Care.

[6] Parikh CR, Mishra J, Thiessen-Philbrook H, Dursun B, Ma Q, Kelly C (2006). Urinary IL-18 is an early predictive biomarker of acute kidney injury after cardiac surgery. Kidney Int.

[7] Mishra J, Ma Q, Prada A, Mitsnefes M, Zahedi K, Yang J (2003). Identification of neutrophil gelatinase-associated lipocalin as a novel early urinary biomarker for ischemic renal injury. J Am Soc Nephrol.

[8] Uchino S, Kellum JA, Bellomo R, Doig GS, Morimatsu H, Morgera S (2005). Acute renal failure in critically ill patients: a multinational, multicenter study. JAMA.

[9] Hoste EA, Clermont G, Kersten A, Venkataraman R, Angus DC, De Bacquer D (2006). RIFLE criteria for acute kidney injury are associated with hospital mortality in critically ill patients: a cohort analysis. Crit Care.

[10] Liangos O, Wald R, O’Bell JW, Price L, Pereira BJ, Jaber BL (2006). Epidemiology and outcomes of acute renal failure in hospitalized patients: a national survey. Clin J Am Soc Nephrol.

[11] Lameire N, Van Biesen W, Vanholder R (2005). Acute renal failure. Lancet.

[12] Schrier RW, Wang W (2004). Acute renal failure and sepsis. N Engl J Med.

[13] Wan L, Bagshaw SM, Langenberg C, Saotome T, May C, Bellomo R (2008). Pathophysiology of septic acute kidney injury: what do we really know?. Crit Care Med.

[14] Hotchkiss RS, Swanson PE, Freeman BD, Tinsley KW, Cobb JP, Matuschak GM (1999). Apoptotic cell death in patients with sepsis, shock, and multiple organ dysfunction. Crit Care Med.

[15] Lerolle N, Nochy D, Guerot E, Bruneval P, Fagon JY, Diehl JL (2010). Histopathology of septic shock induced acute kidney injury: apoptosis and leukocytic infiltration. Intensive Care Med.

[16] Ostermann M, Chang RW (2007). Acute kidney injury in the intensive care unit according to RIFLE. Crit Care Med.

[17] Esson ML, Schrier RW (2002). Diagnosis and treatment of acute tubular necrosis. Ann Intern Med.

[18] Knotek M, Rogachev B, Wang W, Ecder T, Melnikov V, Gengaro PE (2001). Endotoxemic renal failure in mice: Role of tumor necrosis factor independent of inducible nitric oxide synthase. Kidney Int.

[19] Cunningham PN, Dyanov HM, Park P, Wang J, Newell KA, Quigg RJ (2002). Acute renal failure in endotoxemia is caused by TNF acting directly on TNF receptor-1 in kidney. J Immunol.

[20] Bellomo R, Kellum JA, Ronco C (2004). Defining acute renal failure: physiological principles. Intensive Care Med.

[21] Cruz DN, Mehta RL (2014). Acute kidney injury in 2013: Breaking barriers for biomarkers in AKI--progress at last. Nature reviews Nephrology.

[22] Neziri D, Ilhan A, Maj M, Majdic O, Baumgartner-Parzer S, Cohen G (2010). Cloning and molecular characterization of Dashurin encoded by C20orf116, a PCI-domain containing protein. Biochim Biophys Acta.

[23] Wu J, Lei G, Mei M, Tang Y, Li H A novel C53/LZAP-interacting protein regulates stability of C53/LZAP and DDRGK domain-containing Protein 1 (DDRGK1) and modulates NF-kappaB signaling. J Biol Chem.

[24] Mayerhofer M, Gleixner KV, Mayerhofer J, Hoermann G, Jaeger E, Aichberger KJ (2008). Targeting of heat shock protein 32 (Hsp32)/heme oxygenase-1 (HO-1) in leukemic cells in chronic myeloid leukemia: a novel approach to overcome resistance against imatinib. Blood.

[25] Tatsumi K, Sou YS, Tada N, Nakamura E, Iemura S, Natsume T A novel type of E3 ligase for the Ufm1 conjugation system. J Biol Chem.

[26] Lassnigg A, Schmid ER, Hiesmayr M, Falk C, Druml W, Bauer P (2008). Impact of minimal increases in serum creatinine on outcome in patients after cardiothoracic surgery: do we have to revise current definitions of acute renal failure?. Crit Care Med.

[27] Blaikley J, Sutton P, Walter M, Lapsley M, Norden A, Pugsley W (2003). Tubular proteinuria and enzymuria following open heart surgery. Intensive Care Med.

